# Current clinical trials testing the combination of immunotherapy with radiotherapy

**DOI:** 10.1186/s40425-016-0156-7

**Published:** 2016-09-20

**Authors:** Josephine Kang, Sandra Demaria, Silvia Formenti

**Affiliations:** 1 Department of Radiation Oncology, Weill Cornell Medicine, 525 East 68th Street, New York, NY 10065 USA; 2 Department of Radiation Oncology, Stich Radiation Center, 525 East 68th Street, New York, NY 10065 USA

**Keywords:** Antitumor immunity, Immunotherapy, Radiotherapy, Stereotactic body radiation therapy, Abscopal effect, CTLA-4, PD-1, PD-L1, TGFβ

## Abstract

Increasing evidence demonstrates that radiation acts as an immune stimulus, recruiting immune mediators that enable anti-tumor responses within and outside the radiation field. There has been a rapid expansion in the number of clinical trials harnessing radiation to enhance antitumor immunity. If positive, results of these trials will lead to a paradigm shift in the use of radiotherapy. In this review, we discuss the rationale for trials combining radiation with various immunotherapies, provide an update of recent clinical trial results and highlight trials currently in progress. We also address issues pertaining to the optimal incorporation of immunotherapy with radiation, including sequencing of treatment, radiation dosing and evaluation of clinical trial endpoints.

## Introduction

Radiation therapy (RT) is a longstanding pillar of cancer treatment, historically utilized to treat a discrete target and provide local tumor control. Mounting evidence demonstrates that RT also elicits an immune response that can manifest as an immune-mediated tumor regression outside of the targeted site [1]. The potential ability of RT to induce an immunogenic cell death and counteract an immune-suppressive tumor microenvironment to effectively convert the irradiated tumor into an *in situ* vaccine has implications for both local and systemic disease control, and provides the primary rationale for combining novel immunotherapies with RT.

The combination of immunotherapy with RT is an actively growing field of clinical investigation, with rapid expansion in the number and type of clinical trials. After a brief summary on the immunomodulatory properties of radiation, this review will describe some of the available types of immunotherapies that are currently being tested in combination with radiotherapy. We will discuss the challenges in the design and evaluation of current trials, including selection of appropriate radiation dose, fractionation, sequencing of therapies and meaningful trial endpoints.

### Radiation and immune-mediated tumor response

Historically, RT has been considered to be immunosuppressive, based on older treatment techniques with large fields that included substantial bone marrow volume and/or circulating blood volume resulting in reduced blood cell counts [2, 3]. In addition, because of the relative radio-sensitivity of hematopoietic cells, whole-body RT regimens are used prior to stem cell transplantation to cause lympho- and myelo-ablation [4, 5].

To minimize side effects to normal tissue, conventional treatment regimens deliver the radiation dose that is effective at controlling a tumor (40 to 70 Gy, depending on tumor type) in multiple small daily doses, given over several weeks. Advancements in RT equipment and planning systems allow delivery of highly conformal treatment with increased precision, and as a result, it is now possible to deliver higher doses per fraction while sparing adjacent normal tissue. Techniques such as stereotactic radiosurgery and body radiation therapy (SRS, SBRT), intensity modulated radiation therapy (IMRT) and image-guided radiation therapy (IGRT) have transformed delivery of RT, broadening the range of RT applications. Since the effects of radiation on the immune system and on the tumor microenvironment may depend on the dose and delivery methods used, when radiation is used to stimulate an anti-tumor immune response in combination with immunotherapy thoughtful considerations on delivery techniques, dose, and fractionation are warranted.

The traditional model of radiation-induced tumor control that forms the basis for conventional fractionated RT regimens is built on four well-established principles of radiobiology, the fours “Rs”: reassortment of cells to a cell cycle phase that is radiation-sensitive after each fraction; repair of sublethal damage in normal cells to decrease toxicity, reoxygenation of hypoxic tumor regions, and repopulation [6]. In this model, the therapeutic effect of radiation is mainly attributed to direct DNA damage and indirect damage from free radical formation [7]. However, preclinical studies over the past decade have demonstrated an important contribution from the effects of RT on the tumor microenvironment and on the anti-tumor immune response, giving rise to the concept that the fifth “R” of radiobiology is immune-mediated tumor Rejection [8].

The multitude of biological responses elicited by RT that have been shown in experimental models to convert the irradiated tumor into an in situ vaccine have been detailed in several recent reviews [9–11]. Here, we will only briefly highlight a few concepts that more significantly impact clinical trial design. In vitro, immunogenic cell death (ICD) is induced by RT in a dose-dependent way [12], suggesting that larger doses should have more pro-immunogenic effects. However, in vivo data suggest a more complex relationship with dose and fractionation. The ability of RT to induce priming of anti-tumor T cells is influenced by the pre-existing tumor microenvironment, and by the effects of RT on immune cells and other components of the tumor microenvironment [7]. So far, pre-clinical studies have not achieved a consensus about the optimal dose and regimen to be used to stimulate the immune system, with some studies supporting the use a single large doses (e.g., 30 Gy), and others showing that standard dose of 2 Gy or classical hypo-fractionated doses of 6 or 8 Gy delivered daily in consecutive days are more effective [13–15]. Importantly, radiation-induced T cell responses to immunogenic exogenous antigens (e.g., ovalbumin) expressed by tumors at relatively high levels were demonstrated [16]. However, in other experiments induction of T cell responses to shared tumor antigens derived from self-proteins that are over-expressed by cancer cells could not be detected unless mice were given immunotherapy to overcome key immunosuppressive pathways [10]. Thus, while several types of immunotherapy have been shown to work in concert with RT to induce anti-tumor T cell responses, [9] the requirements for a specific combination to be effective, in terms of RT dose and fractionation and sequencing of RT with immunotherapy have not been fully explored in pre-clinical studies, leaving multiple open questions for optimal design of a clinical study.

In the next paragraphs, we will succinctly describe the available immunotherapies, then focus on active clinical trials that are investigating their synergy with radiation.

### Immunotherapy: new therapeutic opportunities

Significant progress in immuno-oncology has led to new treatments such as immune checkpoint blockade, adoptive T cell transfer, cytokine therapy, dendritic cell and peptide vaccines, and monoclonal antibody treatment. Many of these immunotherapies have been tested in combination with RT in pre-clinical studies and are under investigation in the clinic.

Induction of anti-tumor immunity is a multi-step process, which is regulated at every step by positive and negative signals [17]. First, tumor antigens must reach professional antigen-presenting cells (APCs), such as dendritic cells, which upon activation migrate to draining lymph nodes. There, DCs, after processing the peptides in the proteasome, present them as part of the major histocompatibility (MHC) molecules to T cells, in the context of co-stimulatory signals that result in T cell activation and proliferation. Once activated, tumor-specific T cells differentiate into effectors and home to the tumor where they must overcome the existing immune-suppressive microenvironment to successfully reject it. Other T cells differentiate into memory T cells, potentially capable to protect the host from micrometastatic disease that emerges from dormancy and prevent tumor recurrence [18]. Immunotherapies currently approved and in development act at one or more of the steps of this process. RT has been shown to potentially enhance each step, including uptake of tumor antigens by dendritic cells and their activation, as well as migration of the activated effector T cells back to the tumor. Thus RT could enhance and complement the action of many different immunotherapy agents [19–21].

Some of the emerging therapies are summarized below, along with combination approaches incorporating RT.

#### Immune checkpoint blockade

Immune checkpoints trigger downstream signaling pathways that inhibit T cells activation and/or effector function and maintain a balance between stimulation and suppression of immune function, thereby preventing autoimmunity. Antibodies against two immune checkpoints have been approved by FDA for treatment of some cancers.

##### Cytotoxic T-lymphocyte antigen 4 (CTLA-4)

CTLA-4 is an inhibitory receptor that is upregulated after T-cell activation, and transmits signals that suppress T-cell activation and proliferation [22]. It is also expressed at high levels on tumor-infiltrating regulatory T cells (Tregs) and antibodies targeting CTLA-4 can selectively deplete these cells by antibody-mediated cellular cytotoxicity (ADCC) mechanisms [23].

One antibody targeting CTLA-4, ipilimumab (Yervoy, Bristol Myers Squibb), is approved for metastatic melanoma [24]. Tremelimumab is another anti-CTLA-4 antibody currently under development by MedImmune/Astra Zeneca.

A seminal preclinical study demonstrated that RT is synergistic with anti-CTLA antibody and induces systemic anti-tumor responses in a poorly immunogenic carcinoma refractory to anti-CTLA-4 monotherapy [25]. Since that time, there have been multiple case reports and retrospective series reporting abscopal effects in patients receiving RT during treatment with ipilimumab [26–29].

Dose and fractionation schedule likely play an important role in response. Preclinical models support use of hypofractionated RT in daily doses of 6 to 8 Gy given 5 or 3 times, over a single high dose treatment of 20 Gy, in order to maximize likelihood of generating an effective anti-tumor immune response with anti-CTLA-4 [15]. Consistent with this finding, the most dramatic cases of abscopal effect reported with ipilimumab and RT have utilized RT doses and fractionation [26, 27, 29, 30] similar to the RT regimens effective in preclinical studies. However, abscopal effects were also reported in patients treated with a wider range of RT doses [26, 29, 31].

While most abscopal responses reported with addition of RT to ipilimumab were in melanoma, a disease responsive to ipilimumab alone, a notable exception was a patient with metastatic non-small cell lung cancer (NSCLC) resistant to multiple lines of systemic chemotherapy who was treated with ipilimumab and 5 fractions of 6 Gy RT to a single liver metastasis [30]. This resulted in a complete and durable response. A phase 2 study evaluating this regimen (NCT02221739) in NSCLC completed enrollment and confirmed the activity of RT + ipilimumab in a disease that was non-responsive to anti-CTLA-4 alone [32].

A phase 1 dose escalation trial (NCT01497808) evaluated 22 patients with metastatic melanoma, treated with 2-3 fractions of RT to a single lung or osseous metastasis, or 6 Gy to a subcutaneous or hepatic metastasis, followed by ipilimumab [33]. There were no complete responses, though partial responses were noted in 18 % of unirradiated lesions. However, given that ipilimumab at baseline has activity in metastatic melanoma, it is uncertain the degree to which RT contributed to tumor regression.

A phase 1/2 trial enrolled men with metastatic castrate-resistant prostate cancer, and escalated the dose of ipilimumab, with or without concurrent single fraction RT (8 Gy) targeting an osseous metastasis [34]. This approach was further evaluated in a subsequent, multi-institutional, phase 3 trial (NCT00861614) with 799 patients diagnosed with metastatic castrate-resistant prostate cancer, treated with 1 fraction (8 Gy) RT to an osseous metastasis and randomized to ipilimumab or placebo [35]. Primary endpoint of improved overall survival was not achieved, though P-value was close to the cut off for significance (*P* = 0.0530). On subset analysis, patients with non-visceral metastatic disease treated with ipilimumab combined with RT experienced an incremental improvement in overall survival, suggesting non-visceral metastases may be a more appropriate target for RT in combination with ipilimumab. Furthermore, this study demonstrated that the combination of RT and ipilimumab was overall well-tolerated with minimal added toxicity.

Currently ongoing trials combining CTLA-4 inhibition with RT are summarized in Table 1. Metastatic melanoma is the most common primary diagnosis under study, and majority of studies are evaluating RT and CTLA-4 blockade administered in concurrent fashion.

**Table 1:** Active clinical trials combining radiation with CTLA-4 inhibition

NCT Number	Phase	Title	Conditions	Interventions	RT Details	Enrollment	Sponsor/Collaborators
NCT01711515	Phase 1	Chemoradiation Therapy and Ipilimumab in Treating Patients With Locally Advanced Cervical Cancer	Cervical Cancer	Cisplatin, Ipilimumab	EBRT 6 weeks	28	National Cancer Institute (NCI)
NCT02406183	Phase 1	Trial of SBRT With Concurrent Ipilimumab in Metastatic Melanoma	Melanoma	Ipilimumab	SBRT 8-12 Gy × 3 fx	21	Radiotherapie|University Hospital, Ghent
NCT01935921	Phase 1	Ipilimumab, Cetuximab, and Intensity-Modulated Radiation Therapy in Treating Patients With Previously Untreated Stage III-IVB Head and Neck Cancer	Stage III-IVB Head and Neck Cancer	Cetuximab, Ipilimumab	IMRT, 7 wks	18	National Cancer Institute (NCI)
NCT02659540	Phase 1	A Pilot Study to Evaluate the Safety and Efficacy of Combination Checkpoint Blockade Plus External Beam Radiotherapy in Subjects With Stage IV Melanoma	Melanoma	Ipilimumab, Nivolumab	EBRT 3 Gy × 10 fx or 9 Gy × 3 fx	18	Ludwig Institute for Cancer Research|Bristol-Myers Squibb
NCT01860430	Phase 1	A Phase Ib Trial of Concurrent Cetuximab (ERBITUX) and Intensity Modulated Radiotherapy (IMRT) With Ipilimumab (YERVOY) in Locally Advanced Head and Neck Cancer	Head and Neck Cancer	Ipilimumab, Cetuximab	IMRT, 7 wks	18	University of Pittsburgh|National Cancer Institute (NCI)
NCT01996202	Phase 1	A Pilot Study of Ipilumimab and Radiation in Poor Prognosis Melanoma	Melanoma	Ipilimumab	EBRT	24	Duke University
NCT02311361	Phase 1	Immune Checkpoint Inhibition (Tremelimumab and/or MEDI4736) in Combination With Radiation Therapy in Patients With Unresectable Pancreatic Cancer	Pancreatic Cancer	Tremelimumab, MEDI4736	SBRT, 1-5 fx	60	National Cancer Institute (NCI)|National Institutes of Health Clinical Center (CC)
NCT02239900	Phase 1|Phase 2	Ipilimumab and Stereotactic Body Radiation Therapy (SBRT) in Advanced Solid Tumors	Liver cancer, Lung Cancer	Ipilimumab	SBRT 12.5 Gy × 4 fx	120	M.D. Anderson Cancer Center|Bristol-Myers Squibb
NCT02696993	Phase 1|Phase 2	Phase I/II Trial of Nivolumab With Radiation or Nivolumab and Ipilimumab With Radiation for the Treatment of Intracranial Metastases From Non-Small Cell Lung Cancer	Brain Metastases (NSCLC)	Nivolumab, Ipilimumab	WBRT 3 Gy × 10 fx, SRS 1 fx^a^	80	M.D. Anderson Cancer Center|Bristol-Myers Squibb
NCT02254772	Phase 1|Phase 2	TLR9 Agonist SD-101, Ipilimumab, and Radiation Therapy in Treating Patients With Low-Grade Recurrent B-cell Lymphoma	B-cell Lymphoma	Ipilimumab, TLR9 agonist SD-101	RT days 1, 2	27	Ronald Levy|National Cancer Institute (NCI)|Stanford University
NCT01565837	Phase 2	SART: Concurrent Ipilimumab and Stereotactic Ablative Radiation Therapy (SART) for Oligometastatic But Unresectable Melanoma	Melanoma	Ipilimumab	SBRT 1-5 fx	50	Wolfram Samlowski|Comprehensive Cancer Centers of Nevada
NCT01970527	Phase 2	Phase II Trial of Stereotactic Body Radiotherapy Followed by Ipilimumab in Treating Patients With Stage IV Melanoma	Recurrent/Stage IV Melanoma	Ipilimumab	SBRT 3 fx	40	
NCT02107755	Phase 2	Stereotactic Radiation Therapy and Ipilimumab in Treating Patients With Metastatic Melanoma	Melanoma	Ipilimumab	SBRT	32	Ohio State University Comprehensive Cancer Center|Bristol-Myers Squibb
NCT02097732	Phase 2	Ipilimumab Induction in Patients With Melanoma Brain Metastases Receiving Stereotactic Radiosurgery	Brain Metastases (Melanoma)	Ipilimumab	SRS 1 fx^a^	40	University of Michigan Cancer Center
NCT02115139	Phase 2	GRAY-B: GEM STUDY: Radiation And Yervoy in Patients With Melanoma and Brain Metastases	Melanoma	Ipilimumab	WBRT 3 Gy × 10 fx	66	Grupo Espanol Multidisciplinar de Melanoma|Bristol-Myers Squibb
NCT02701400	Phase 2	Tremelimumab and Durvalumab With or Without Radiation Therapy in Patients With Relapsed Small Cell Lung Cancer	Recurrent Small Cell Lung Cancer	Tremelimumab, Durvalumab	SBRT	20	Emory University|AstraZeneca
NCT02434081	Phase 2	NICOLAS: NIvolumab COnsolidation After Standard First-line Chemotherapy and Radiotherapy in Locally Advanced Stage IIIA/B NSCLC	NSCLC	Nivolumab	EBRT	43	European Thoracic Oncology Platform|Bristol-Myers Squibb|Frontier Science Foundation, Hellas
NCT01449279	Pilot	Pilot Ipilimumab in Stage IV Melanoma Receiving Palliative Radiation Therapy	Stage IV Melanoma	Ipilimumab	Palliative RT	20	Stanford University

^a^Dose determined by treating physician

Abbreviations: *Fx* fraction, *EBRT* external beam radiation (standard fractionation), *SBRT* stereotactic body radiation therapy, *SRS* stereotactic radiosurgery, *WBRT* whole brain radiation therapy, *NSCLC* non-small cell lung cancer

##### Programmed death-1 (PD-1)/Programmed death ligand -1(PD-L1)

PD-1 is an inhibitory cell surface receptor that acts as an immune checkpoint. Its ligand, PD-L1 is expressed on diverse types of cells, including antigen-presenting cells, epithelial and endothelial cells. Studies have shown that PD-1 and PD-L1 targeted therapies have clinical activity against metastatic bladder cancer, head and neck cancers, Hodgkin’s lymphoma, non-small cell lung cancer, and renal cell cancer [36]. Anti-PD-1 therapies, pembrolizumab (MK-3475/Keytruda, Merck & Co) and nivolumab (Opdivo, Bristol-Meyers Squibb Co), are currently approved as treatment for unresectable or metastatic melanoma, and as second-line treatment for non-small cell lung cancer (NSCLC) after chemotherapy. Nivolumab has been recently approved for Hodgkin’s lymphoma that has relapsed or progressed after autologous hematopoietic stem cell transplantation, and metastatic renal cell carcinoma. Atezolizumab (MPDL3280A) and durvalumab (MEDI-4736) are monoclonal anti PD-L1 antibodies currently under active investigation in clinical trials. Atezolizumab has been recently approved by FDA for locally advanced or metastatic urothelial carcinoma progressing during or after platinum chemotherapy. REGN2810 is a monoclonal antibody that binds to PD-1 and inhibits PD-L1 mediated activation of downstream pathways.

Preclinical studies demonstrate that the combination of RT and targeted PD-1/PD-L1 therapy activates cytotoxic T-cells, reduces myeloid-derived suppressor cells and induces an abscopal response [14, 37, 38]. Based on these promising results, numerous ongoing clinical trials are testing the combination of PD-1/PD-L1 inhibition and RT (Table 2). Majority of studies are phase 1 or 2. There are two open phase 3 trials looking at combination of nivolumab with RT in locally advanced NSCLC (NCT02768558), and glioblastoma (NCT02617589), respectively; results are pending.

**Table 2:** Active clinical trials combining radiation with PD-1/PD-L1 targeted therapy

NCT Number	Phase	Title	Conditions	RT Details	Enrollment	Sponsor/Collaborators
NCT02642809	Phase 0	Pembrolizumab With Locally Delivered Radiation Therapy for the Initial Treatment of Metastatic Esophageal Cancers	Esophageal Cancer	Hypofx brachytherapy	15	Washington University School of Medicine|Merck Sharp & Dohme Corp.
NCT02463994	Phase 0	A Pilot Study of MPDL3280A and HIGRT in Metastatic NSCLC	NSCLC	Hypofx RT	12	University of Michigan Cancer Center|University of Washington
NCT02587455	Phase 1	Pembrolizumab and Palliative Radiotherapy in Lung	Thoracic Tumours	Palliative EBRT	48	Royal Marsden NHS Foundation Trust|Merck Sharp & Dohme Corp.
NCT02608385	Phase 1	Study of PD1 Blockade by Pembrolizumab With Stereotactic Body Radiotherapy in Advanced Solid Tumors	NSCLC	SBRT, 3-5 fx	138	University of Chicago
NCT02621398	Phase 1	Pembrolizumab, Paclitaxel, Carboplatin, and Radiation Therapy in Treating Patients With Stage II-IIIB NSCLC	NSCLC	EBRT	30	Rutgers, The State University of New Jersey|National Cancer Institute (NCI)|Merck Sharp & Dohme Corp.
NCT02313272	Phase 1	Phase I Trial of Hypofractionated Stereotactic Irradiation (HFSRT) With Pembrolizumab and Bevacizumab for Recurrent High Grade Gliomas	Malignant Glioma	FSRT over 5 days	32	H. Lee Moffitt Cancer Center and Research Institute|Merck Sharp & Dohme Corp.
NCT02402920	Phase 1	Phase I Trial of MK-3475 and Concurrent Chemo/Radiation for the Elimination of Small Cell Lung Cancer	Lung Cancer	EBRT, 1.5 Gy × 30 fx, BID	80	M.D. Anderson Cancer Center|Merck Sharp & Dohme Corp.
NCT02716948	Phase 1	Stereotactic Radiosurgery and Nivolumab in Treating Patients With Newly Diagnosed Melanoma Metastases in the Brain or Spine	Melanoma (Brain metastases)	SRS	90	Sidney Kimmel Comprehensive Cancer Center
NCT02560636	Phase 1	Pembrolizumab in Muscle Invasive/Metastatic Bladder Cancer	Invasive Bladder Cancer	Hypofx RT	34	Royal Marsden NHS Foundation Trust|Merck Sharp & Dohme Corp.
NCT02318771	Phase 1	Radiation Therapy and MK-3475 for Patients With Recurrent/Metastatic Head and Neck Cancer, Renal Cell Cancer, Melanoma, and Lung Cancer	Metastatic HN, RCC, Melanoma, Lung Cancer	EBRT	40	Thomas Jefferson University|Merck Sharp & Dohme Corp.
NCT02648633	Phase 1	Stereotactic Radiosurgery With Nivolumab and Valproate in Patients With Recurrent Glioblastoma	Glioblastoma	SRS	17	University of Virginia
NCT02303366	Phase 1	Pilot Study of Stereotactic Ablation for Oligometastatic Breast Neoplasia in Combination With the Anti-PD-1 Antibody MK-3475	Oligometastatic Breast Cancer	SBRT, 20 Gy × 1 fx	15	Peter MacCallum Cancer Centre, Australia
NCT02659540	Phase 1	A Pilot Study to Evaluate the Safety and Efficacy of Combination Checkpoint Blockade Plus External Beam Radiotherapy in Subjects With Stage IV Melanoma	Melanoma	3 Gy × 10 fx, or 9 Gy × 3 fx	18	Ludwig Institute for Cancer Research|Bristol-Myers Squibb
NCT02586207	Phase 1	Pembrolizumab in Combination With CRT for LA-SCCHN	Advanced HN Cancers	EBRT, 7 wks	39	Sanford Health|Merck Sharp & Dohme Corp.
NCT02303990	Phase 1	RADVAX: A Stratified Phase I Trial of Pembrolizumab With Hypofractionated Radiotherapy in Patients With Advanced and Metastatic Cancers	Metastatic Cancers	Hypofx RT	70	Abramson Cancer Center of the University of Pennsylvania
NCT02764593	Phase 1	Chemotherapy +/- Nivolumab in Patients With Intermediate and High-Risk Local-Regionally Advanced Head and Neck Squamous Cell Carcinoma	Advanced HN Cancers	IMRT, 7 wks	120	RTOG Foundation, Inc.|Bristol-Myers Squibb
NCT02400814	Phase 1	MPDL3280A and Stereotactic Ablative Radiotherapy in Patients With NSCLC	NSCLC	SBRT, 5 fx	45	University of California, Davis|National Cancer Institute (NCI)|Genentech, Inc.
NCT02383212	Phase 1	Study of REGN2810 (Anti-PD-1) in Patients With Advanced Malignancies	Advanced Cancer	Hypofx RT	973	Regeneron Pharmaceuticals|Sanofi
NCT02311361	Phase 1	Immune Checkpoint Inhibition (Tremelimumab and/or MEDI4736) in Combination With Radiation Therapy in Patients With Unresectable Pancreatic Cancer	Pancreatic Cancer	SBRT, 1-5 fx	60	National Cancer Institute (NCI)|National Institutes of Health Clinical Center (CC)
NCT02696993	Phase 1|Phase 2	Phase I/II Trial of Nivolumab With Radiation or Nivolumab and Ipilimumab With Radiation for the Treatment of Intracranial Metastases From NSCLC	Lung Cancer (Brain metastases)	WBRT 3 Gy × 10 fx, SRS 1 fx	80	M.D. Anderson Cancer Center|Bristol-Myers Squibb
NCT02444741	Phase 1|Phase 2	MK-3475 and Hypofractionated Stereotactic Radiation Therapy in Patients With NSCLC (NSCLC)	Lung Cancer	EBRT or SBRT	104	M.D. Anderson Cancer Center|Merck Sharp & Dohme Corp.
NCT02530502	Phase 1|Phase 2	Radiation Therapy With Temozolomide and Pembrolizumab in Treating Patients With Newly Diagnosed Glioblastoma	Glioblastoma	EBRT	50	Northwestern University|Merck Sharp & Dohme Corp.|National Cancer Institute (NCI)
NCT02730546	Phase 1|Phase 2	Pembrolizumab, Combination Chemotherapy, and Radiation Therapy Before Surgery in Treating Adult Patients With Locally Advanced Gastroesophageal Junction or Gastric Cardia Cancer That Can Be Removed by Surgery	Gastric Cancer	EBRT	68	Mayo Clinic|National Cancer Institute (NCI)
NCT02759575	Phase 1|Phase 2	A Study of Chemoradiation Plus Pembrolizumab for Locally Advanced Laryngeal Squamous Cell Carcinoma	Advanced HN Cancers	EBRT	47	Nooshin Hashemi-Sadraei|Merck Sharp & Dohme Corp.|University of Cincinnati
NCT02407171	Phase 1|Phase 2	Evaluating the Combination of MK-3475 and Sterotactic Body Radiotherapy in Patients With Metastatic Melanoma or NSCLC	Melanoma, Lung Cancer	SBRT, 1-5 fx	60	Yale University
NCT02735239	Phase 1|Phase 2	Study of Anti-PD-L1 in Combination With Chemo(Radio)Therapy for Oesophageal Cancer	Esophageal Cancer	EBRT	75	Ludwig Institute for Cancer Research|AstraZeneca
NCT02305186	Phase 1|Phase 2	Safety and Immunological Effect of Pembrolizumab in Resectable or Borderline Resectable Pancreatic Cancer	Pancreatic Cancer	EBRT, 1.8 Gy × 28 fx	56	Osama Rahma, MD|M.D. Anderson Cancer Center|University of Virginia
NCT02599779	Phase 2	A Proof of Principle Study of Pembrolizumab With SBRT in TKI mRCC Patients	Metastatic RCC	SBRT	35	Sunnybrook Health Sciences Centre|Merck Sharp & Dohme Corp.|Ozmosis Research Inc.
NCT02648282	Phase 2	Study With CY, Pembrolizumab, GVAX, and SBRT in Patients With Locally Advanced Pancreatic Cancer	Pancreatic Cancer	SBRT, 3-5 fx	54	Sidney Kimmel Comprehensive Cancer Center|Merck Sharp & Dohme Corp.
NCT02492568	Phase 2	Pembrolizumab After SBRT Versus Pembrolizumab Alone in Advanced NSCLC	NSCLC	SBRT, 8 Gy × 3 fx	74	The Netherlands Cancer Institute|Merck Sharp & Dohme Corp.
NCT02641093	Phase 2	Phase II Trial of Adjuvant Cisplatin and Radiation With Pembrolizumab in Resected Head and Neck Squamous Cell Carcinoma	HN Cancer	EBRT, 6 wks	80	Trisha Wise-Draper|Merck Sharp & Dohme Corp.|University of Cincinnati
NCT02684253	Phase 2	Screening Trial of Nivolumab With Image Guided, Stereotactic Body Radiotherapy (SBRT) Versus Nivolumab Alone in Patients With Metastatic Head and Neck Squamous Cell Carcinoma (HNSCC)	HN Cancer	SBRT, 9 Gy x 3 fx	40	Memorial Sloan Kettering Cancer Center|University of Chicago
NCT02667587	Phase 2	Study of Temozolomide Plus Radiation Therapy With Nivolumab or Placebo, for Newly Diagnosed Patients With Glioblastoma (GBM, a Malignant Brain Cancer).	Glioblastoma	EBRT	320	Bristol-Myers Squibb|Ono Pharmaceutical Co. Ltd
NCT02437071	Phase 2	Assess the Efficacy of Pembrolizumab Plus Radiotherapy or Ablation in Metastatic Colorectal Cancer Patients	Metastatic Colorectal Cancer	EBRT vs radiofrequency ablation	48	Memorial Sloan Kettering Cancer Center|Merck Sharp & Dohme Corp.
NCT02658097	Phase 2	A Randomized Two Arm Phase II Trial of Pembrolizumab Alone or Sequentially Following Single Fraction Non-ablative Radiation to One of the Target Lesions, in Previously Treated Patients With Stage IV NSCLC	Stage IV NSCLC	8 Gy × 1 fx	66	Case Comprehensive Cancer Center
NCT02562625	Phase 2	Trial of Pembrolizumab and Radiotherapy in Melanoma	Melanoma	8 Gy × 3 fx	234	Royal Marsden NHS Foundation Trust|University of Manchester|University of Leeds
NCT02730130	Phase 2	Study to Assess the Efficacy of Pembrolizumab Plus Radiotherapy in Metastatic Triple Negative Breast Cancer Patients	Metastatic Breast Cancer	EBRT	17	Memorial Sloan Kettering Cancer Center|Merck Sharp & Dohme Corp.
NCT02707588	Phase 2	Tolerance and Efficacy of Pembrolizumab or Cetuximab Combined With RT in Patients With Locally Advanced HNSCC	Advanced HN Cancers	EBRT	114	Groupe Oncologie Radiotherapie Tete et Cou
NCT02621151	Phase 2	Pembrolizumab (MK3475), Gemcitabine, and Concurrent Hypofractionated Radiation Therapy for Muscle-Invasive Urothelial Cancer of the Bladder	Muscle-invasive Urothelial Cancer of the Bladder	Hypofx RT	54	New York University School of Medicine|Merck Sharp & Dohme Corp.
NCT02609503	Phase 2	Pembrolizumab + Radiation for Locally Adv SCC of the Head and Neck (SCCHN) Not Eligible Cisplatin	Head and Neck Cancer	IMRT, 7 wks	29	UNC Lineberger Comprehensive Cancer Center|Merck Sharp & Dohme Corp.
NCT02677155	Phase 2	Sequential Intranodal Immunotherapy (SIIT) Combined With Anti-PD1 (Pembrolizumab) in Follicular Lymphoma	Follicular Lymphoma	8 Gy × 1 fx	20	Oslo University Hospital|Norwegian Cancer Society|Merck Sharp & Dohme Corp.
NCT02586610	Phase 2	Trial of Chemoradiation and Pembrolizumab in Patients With Rectal Cancer	Rectal Cancer	EBRT, 1.8 Gy × 28 fx	53	Osama Rahma, MD|Hoosier Cancer Research Network|Merck Sharp & Dohme Corp.
NCT02434081	Phase 2	NIvolumab COnsolidation After Standard First-line Chemotherapy and Radiotherapy in Locally Advanced Stage IIIA/B NSCLC	NSCLC	EBRT	43	European Thoracic Oncology Platform|Bristol-Myers Squibb|Frontier Science Foundation, Hellas
NCT02296684	Phase 2	Immunotherapy With MK-3475 in Surgically Resectable Head and Neck Squamous Cell Carcinoma	HN Cancer	IMRT, 6 wks	46	Washington University School of Medicine|Merck Sharp & Dohme Corp.
NCT02499367	Phase 2	Nivolumab After Induction Treatment in Triple-negative Breast Cancer (TNBC) Patients	Breast Cancer	20 Gy × 1, or 8 Gy × 3	84	The Netherlands Cancer Institute|Bristol-Myers Squibb
NCT02635360	Phase 2	Pembrolizumab and Chemoradiation Treatment for Advanced Cervical Cancer	Cervical Cancer	EBRT + brachytherapy	88	Linda R Duska|Merck Sharp & Dohme Corp.|University of Virginia
NCT02662062	Phase 2	Pembrolizumab With Chemoradiotherapy as Treatment for Muscle Invasive Bladder Cancer	Bladder Cancer	EBRT, 6 wks	30	Australian and New Zealand Urogenital and Prostate Cancer Trials Group
NCT02336165	Phase 2	Phase 2 Study of MEDI4736 in Patients With Glioblastoma	Glioblastoma	EBRT	108	Ludwig Institute for Cancer Research|MedImmune LLC
NCT02289209	Phase 2	Reirradiation With MK-3475 (Pembrolizumab) in Locoregional Inoperable Recurrence or Second Primary Squamous Cell CA of the Head and Neck	Recurrent HN Cancers	EBRT	48	Dan Zandberg|Merck Sharp & Dohme Corp.|University of Maryland
NCT02768558	Phase 3	Cisplatin and Etoposide Plus Radiation Followed By Nivolumab/Placebo For Locally Advanced NSCLC	NSCLC	EBRT (IMRT or 3D CRT)	660	RTOG Foundation, Inc.|Bristol-Myers Squibb
NCT02617589	Phase 3	Study of Nivolumab Versus Temozolomide, Given With Radiation Therapy, for Newly-diagnosed Patients With Glioblastoma (GBM, a Malignant Brain Cancer)	Brain Cancer	EBRT	550	Bristol-Myers Squibb|Ono Pharmaceutical Co. Ltd

Abbreviations: *3D CRT* 3-dimensional conformal radiation therapy, *EBRT* external beam radiation in conventional fractions, *fx* fractions, *hypofx* hypofractionated, *IMRT* intensity modulated radiation therapy, *FSRT* fractionated stereotactic radiation, *HN* head and neck, *SBRT* stereotactic body radiation therapy, *SRS* stereotactic radiosurgery

#### Transforming growth factor beta (TGFβ) targeting

TGFβ is a cytokine with immunosuppressive activity that is activated by RT in the tumor microenvironment [39–41]. Preclinical studies have shown that inhibition of TGFb during and after RT allows priming of T cells to multiple tumor antigens leading to immune-mediated regression of the irradiated tumor and non-irradiated metastases [41]. A few clinical studies are ongoing to test the benefits of TGFβ inhibition and RT (Table 3). One phase 1/2 trial in patients with metastatic breast tested the use of a neutralizing antibody, fresolimumab, with RT and is closed to accrual (NCT01401062). A second trial in the same patient population is accruing and uses a small molecule inhibitor, LY2157299, to target the TGFb receptor (NCT02538471). Fresolimumab is also being tested in the SABR-ATAC phase 1/2 trial in patients with stage IA/IB non-small cell lung cancer with the primary endpoint of evaluating late RT-induced fibrosis inhibition in the phase 2 study (NCT02581787). Another phase 2 study testing LY2157299 in rectal cancer in combination with chemotherapy and RT is not yet open (NCT02688712). A phase 1b/2a study in testing chemoRT and LY2157299 in glioma patients has completed accrual (NCT01220271).

**Table 3:** Active clinical trials combining radiation with TGF-β blockade or GM-CSF

NCT Number	Phases	Title	Condition	Intervention	RT Details	Enrollment	Sponsor/Collaborators
Anti-TGF- β							
NCT02538471	Phase 2	LY2157299 Monohydrate (LY2157299) and Radiotherapy in Metastatic Breast Cancer	Metastatic Breast Cancer	LY2157299	RT, 7.5 Gy × 3 fx	28	Weill Medical College of Cornell University|University of California, Los Angeles
NCT02581787	Phase 1|Phase 2	SABR-ATAC: A Trial of TGF-beta Inhibition and Stereotactic Ablative Radiotherapy for Early Stage Non-small Cell Lung Cancer	Stage IA-B NSCLC	Fresolimumab	SBRT, 4 fx	60	Maximilian Diehn|National Cancer Institute (NCI)|Stanford University
GM-CSF							
NCT02623595	Phase 2	A Study of SBRT in Combination With rhGM-CSF for Stage IV NSCLC Patients Who Failed in Second-line Chemotherapy	Non-small cell lung cancer	GM-CSF	SBRT, 10 Gy × 5 fx	60	Wuhan University|Tongji Hospital|Hubei Cancer Hospital
NCT02663440	Phase 2	Trial of Hypofractionated Intensity Modulated Radiation Therapy With Temozolomide and Granulocyte-macrophage Colony-stimulating Factor for Patients With Newly Diagnosed Glioblastoma Multiforme	Glioblastoma	GM-CSF, Temozolomide	Hypofx IMRT	41	Zhejiang Cancer Hospital
NCT02677155	Phase 2	Sequential Intranodal Immunotherapy (SIIT) Combined With Anti-PD1 (Pembrolizumab) in Follicular Lymphoma	Follicular Lymphoma	GM-CSF, Pembrolizumab, Rituximab, autologous dendritic cells	8 Gy × 1	20	Oslo University Hospital|Norwegian Cancer Society|Merck Sharp & Dohme Corp.
NCT02648282	Phase 2	Study With CY, Pembrolizumab, GVAX, and SBRT in Patients With Locally Advanced Pancreatic Cancer	Pancreatic Cancer	GVAX, Cyclophosphamide, Pembrolizumab	SBRT, 6.6 Gy × 5 fx	54	Sidney Kimmel Comprehensive Cancer Center|Merck Sharp & Dohme Corp.

Abbreviations: *GM-CSF* granulocyte macrophage colony stimulating factor, *Hypofx* hypofractionated, *IMRT* intensity modulated radiation therapy, *NSCLC* non-small cell lung cancer, *SBRT* stereotactic body radiation therapy

#### Cytokines

##### Granulocyte macrophage colony-stimulating factor (GM-CSF)

GM-CSF is a growth factor secreted by macrophages, T-cells, NK cells, endothelial cells and fibroblasts, promoting maturation of dendritic cells and enabling cross-presentation of tumor cell antigens to generate memory T-cells. Preclinical data supports the combination of RT and cytokines to generate an abscopal effect [42]. Based on this, a proof-of-principle pilot study was initiated with 10 fractions of RT (3.5 Gy per fraction) directed to a metastatic lesion and 14 days of GM-CSF. Out of 41 patients with varied tumor histologies, 11 (26.8 %) exhibited clinical response outside of the RT field [43].

GM-CSF gene-transduced tumor cells have shown promise as vaccines (GVAX), demonstrating durable tumor regression in clinical trials enrolling patients with advanced NSCLC, and reports of tumor regression in patients with renal cell carcinoma and melanoma [44]. Currently ongoing trials utilizing GM-CSF or GVAX with radiation are summarized in Table 3. There are far fewer studies for GM-CSF in combination with RT in comparison to immune check point inhibitors.

##### Interleukin-2 (IL-2)

IL-2 is a cytokine produced by T-cells that stimulates T-cell proliferation and antigen-specific differentiation. Treatment with high-dose IL-2 has been documented to induce a complete response in patients with metastatic renal cell carcinoma (RCC), and promote tumor regression in melanoma, but only a small percentage of patients respond [45, 46]. Preclinical data suggests that RT can promote memory T-cell formation through release of cytokines and upregulation of MHC-1, [47, 48] providing rationale for a phase 1 study that combined 1-3 fractions of RT (20 Gy per fraction) with high dose IL-2 for patients with metastatic RCC and melanoma. Response to treatment was correlated with a statistically significant increase in proliferating early activated effector memory T-cells [49]. Although IL-2 has shown promising potential in a subset of patients, there are significant toxicities associated with treatment, ranging from flu-like symptoms to vascular leak syndrome, which is the most frequent and severe complication of treatment [50, 51].

Currently, four phase 2 clinical trials are investigating the combination of high-dose IL-2 with SBRT in 1-3 fractions of 6-20 Gy each, enrolling patients with metastatic melanoma or RCC (Table 4). There are also phase 1 and 2 trials evaluating use of a recombinant fusion protein consisting of IL-2 with L19 human vascular targeting antibody (Darleukin, L19-IL2), which promotes lymphocyte and NK cell stimulation and immune-mediated tumor cell death, with less toxicity [52–55]. This is delivered together with ablative doses of radiation in patients with oligometastatic or limited metastatic disease. Ongoing trials are summarized in Table 4.

**Table 4:** Active clinical trials combining radiation, miscellaneous

NCT Number	Phase	Title	Conditions	Interventions	RT Details	Enrollment	Sponsor/Collaborators
Interleukin 2							
NCT02086721	Phase 1	Phase I Clinical Study Combining L19-IL2 With SABR in Patients With Oligometastatic Solid Tumor	Solid Tumour	L19-IL-2 (recombinant protein with IL-2)	SBRT, 7.5-30 Gy in 1-8 fx	18	Maastricht Radiation Oncology
NCT01416831	Phase 2	Comparison of High-dose IL-2 and High-dose IL-2 With Radiation Therapy in Patients With Metastatic Melanoma.	Metastatic Melanoma	IL-2	SBRT, 20 Gy × 1-2 fx	44	Providence Health & Services|Prometheus
NCT01896271	Phase 2	High Dose IL-2 and Stereotactic Ablative Body Radiation Therapy for Metastatic Renal Cancer	Metastatic RCC, Melanoma	IL-2	SBRT, 8-20 Gy in 1-3 fx	26	University of Texas Southwestern Medical Center|Prometheus
NCT01884961	Phase 2	Radiotherapy as an Immunological Booster in Patients With Metastatic Melanoma or Renal Cell Carcinoma Treated With High-dose Interleukin-2	Metastatic RCC, Melanoma	IL-2	SBRT, 6-12 Gy × 3 fx	19	Istituto Scientifico Romagnolo per lo Studio e la cura dei Tumori
NCT02306954	Phase 2	Study of High Dose Interleukin-2 (IL-2) and Stereotactic Body Radiation (SBRT) in Patients With Metastatic Renal Cancer	Renal Cell Carcinoma	IL-2	SBRT, 20 Gy × 2 fx	84	Providence Health & Services|Prometheus Laboratories
NCT02735850	Phase 2	Combination of SABR and L19-IL2 in Patients With Stage IV Lung Cancer (ImmunoSABR)	Stage IV NSCLC, Limited Metastatic Disease	L19-IL-2	SBRT	141	Maastricht Radiation Oncology
Other Cytokines							
NCT01973322	Phase 2	Vaccination With Autologous Dendritic Cells Loaded With Autologous Tumor Lysate or Homogenate Combined With Immunomodulating Radiotherapy and/or Preleukapheresis IFN-alfa in Patients With Metastatic Melanoma: a Randomized “Proof-of-principle” Phase II Study (ABSIDE)	Metastatic Melanoma	IFN-alfa	IMRT, 8-12 Gy in 3 fx	24	Istituto Scientifico Romagnolo per lo Studio e la cura dei Tumori
OX40 agonists							
NCT01862900	Phase 1|Phase 2	Phase I/II Study of Stereotactic Body Radiation Therapy to Metastatic Lesions in the Liver or Lung in Combination With Monoclonal Antibody to OX40 (MEDI6469) in Patients With Progressive Metastatic Breast Cancer After Systemic Therapy	Metastatic Breast Cancer	OX40 antibody (MEDI6469)	SBRT, 10-25 Gy in 1-2 fractions	40	Providence Health & Services
TLR agonists							
NCT02180698	Phase 1	TLR4 Agonist GLA-SE and Radiation Therapy in Treating Patients With Soft Tissue Sarcoma That Is Metastatic or Cannot Be Removed by Surgery	Metastatic Sarcoma	TLR-4 agonist (GLA-SE)	RT, 5-6 fx over 2 wks	18	Fred Hutchinson Cancer Research Center
NCT02061449	Phase 1	Poly ICLC, Radiation, and Romidepsin for Advanced Cutaneous T Cell Lymphoma	Cutaneous T-cell Lymphoma	TLR3 agonist Poly-ICLC	RT, 3 fx	24	New York University School of Medicine|Ludwig Institute for Cancer Research
NCT02254772	Phase 1|Phase 2	TLR9 Agonist SD-101, Ipilimumab, and Radiation Therapy in Treating Patients With Low-Grade Recurrent B-cell Lymphoma	Recurrent Lymphoma	TLR-9 agonist (SD-101), Ipilimumab	local RT	27	Ronald Levy|National Cancer Institute (NCI)|Stanford University
NCT01421017	Phase 1|Phase 2	Toll-like Receptor (TLR) 7 Agonist, Cyclophosphamide, and Radiotherapy for Breast Cancer With Skin Metastases	Metastatic Breast Cancer	TLR-7 agonist Imiquimod, Cyclophosphamide	RT, 6 Gy × 5 fx	55	New York University School of Medicine|National Cancer Institute (NCI)
NCT01976585	Phase 1|Phase 2	In Situ Vaccine for Low-Grade Lymphoma: Combination of Intratumoral Flt3L and Poly-ICLC With Low-Dose Radiotherapy	Lymphoma	CDX-301, Poly-ICLC (TLR agonist)	Low dose RT	30	Joshua Brody|Icahn School of Medicine at Mount Sinai
Cancer Vaccines							
NCT01436968	Phase 3	Phase 3 Study of ProstAtak™ Immunotherapy With Standard Radiation Therapy for Localized Prostate Cancer	Prostate cancer	AdV-tk vs placebo, valacyclovir +/- ADT	EBRT, 8 wks	711	Advantagene
NCT02446093	Phase 1|Phase 2	Neoadjuvant GMCI Plus mFOLFIRINOX and Chemoradiation for Non-Metastatic Pancreatic Adenocarcinoma (PaTK02)	Pancreatic cancer	AdV-tk, mFOLFIRINOX, gemcitabine	EBRT	44	Ohio State University
NCT01833208		Radiation Therapy in Treating Patients With Metastatic Hormone-Resistant Prostate Cancer Receiving Sipuleucel-T	Metastatic Prostate Cancer	Sipuleucel-T	High-dose 1 fx to bony metastasis	15	Roswell Park Cancer Institute|National Cancer Institute (NCI)|Dendreon
NCT01818986	Phase 2	Sipuleucel-T and Stereotactic Ablative Body Radiation (SABR) for Metastatic Castrate-resistant Prostate Cancer (mCRPC)	Metastatic Prostate Cancer	Sipuleucel-T	SBRT, metastatic site	41	University of Texas Southwestern Medical Center
NCT02232230	Phase 2	A Multicenter Trial Enrolling Men With Advanced Prostate Cancer Who Are to Receive Combination Radiation and Sipuleucel-T	Metastatic Prostate Cancer	Sipuleucel-T	RT, metastatic site	100	21st Century Oncology|Dendreon

##### Interferon alpha (IFN-α)

Interferon alpha (IFN-α) generated interest after preliminary studies demonstrated a survival advantage in pancreatic cancer patients, resulting in a phase 2 trial (NCT00059826) combining IFN-α with adjuvant chemoRT in resected pancreatic cancer patients [56]. Although the observed median survival of 25.4 months was promising compared to historical standards, the regimen had considerable high-grade toxicity, resulting in early closure of the study.

Results of a phase 2 trial (NCT01276730) randomizing locally advanced cervical cancer patients to RT with cisplatin versus IFN-α plus retinoic acid were recently reported [57]. Primary endpoint was overall survival at 3 years. Unfortunately, there was no survival advantage demonstrable with the addition of IFN-α, though toxicity was acceptable. There is currently one actively recruiting phase 2 study (ABSIDE, NCT01973322), randomizing patients with metastatic melanoma to four different arms, with one of the arms exploring use of IFNα with a dendritic cell vaccine and RT, 8-12 Gy in 3 fractions; results are pending.

##### Tumor necrosis factor alpha (TNF-α)

TNF-α is a cytokine produced by activated macrophages and other immune cells, inducing diverse effects such as immune cell activation and tumor cell apoptosis. Initial studies incorporating TNF-α with radiation resulted in unacceptably high rates of toxicity [58]. TNFerade is an adenovector-based gene therapy agent, wich allows radiation-induced translation of TNF- α via a radiation-inducible promoter. Studies using TNFerade intratumoral injection with RT showed acceptable toxicity, [59] providing stimulus for subsequent phase 1 and 2 studies exploring the combination of TNFerade with RT in different primary cancers. Phase 1 studies suggested improved overall and progression-free survival in esophageal cancer, head and neck cancer and other solid tumors [60–64]. This resulted in a phase 3 randomized trial for locally advanced pancreatic cancer patients, treated with standard of care (SOC) chemoRT, vs SOC + TNFerade [65]. Levels of grade 3-4 toxicity were similar between the two arms, but there was no improvement in median survival with the addition of TNFerade. This dampened enthusiasm for this novel approach, and there are currently no open studies exploring TNFerade with RT.

#### Costimulatory antibodies

##### OX40 agonists

OX40 (CD134) is a member of the TNF receptor superfamily, expressed on activated CD4+ and CD8+ T-cells, and induced on activated regulatory T-cells, NK cells and neutrophils [66]. Stimulation of OX40 results in proliferation of CD4+, CD8+ T cells, cytokine production and memory T cell promotion, and suppression of regulatory T-cells, making it an attractive target for combination strategies with RT. Preclinical studies combining single fraction RT (20 Gy) with OX40 activation have demonstrated increased survival and disease regression, correlating with increased tumor infiltration by CD8+ T-cells [67, 68]. The ability of OX40 to enhance T-cell memory and proliferation, while suppressing regulatory T-cell function in preclinical models resulted in enthusiasm for the combination of OX40 agonists with RT, surgery or systemic agents [69]. A phase 1 clinical trial testing an antibody agonist of OX40 with cyclophosphamide and single fraction RT (8 Gy) in metastatic prostate cancer patients is currently ongoing, though no longer recruiting (NCT01642290). Results of the immunological analysis were reported in abstract form in 2013, [70] and showed increased proliferation of CD4+, CD8+ and NK cells in peripheral blood lymphocytes, along with increased interferon gamma and IL-2. There was no change in proliferation of regulatory T-cells. There is also a phase 1/2 trial combining monoclonal antibody agonist of OX40 (MEDI6469) with SBRT in 1-2 fractions of 10-25 Gy each in breast cancer patients with lung or liver metastases (NCT01862900).

#### Dendritic cells (DCs)

The first category of DC-based immunotherapy entails intratumoral injection of autologous DCs to promote the cross priming of T cells to tumor antigens after RT, and increase tumor infiltration of CD8+ T-cells [71]. This approach has been utilized in a phase 1 trial in patients with hepatocellular carcinoma who were treated with single fraction RT (8 Gy) and injected with autologous DCs two days later. Out of 14 patients, 50 % demonstrated a minor to partial clinical response [72]. Another phase I study (NCT00365872) evaluated intratumoral DC injection with neoadjuvant RT to 50 Gy in 25 fractions in soft tissue sarcoma, with primary endpoint to evaluate immunologic response [73]. Ten of 18 patients (56 %) demonstrated a tumor specific immune response, though any correlation with outcome could not be established due to small numbers. The same institution subsequently initiated a phase 2 trial combining intratumoral DC injection and RT to 50 Gy (NCT01347034), which is active but no longer recruiting. There is also a completed phase 1/2 trial with results pending, combining intratumoral DC injection with SBRT and gemcitabine. Currently, there are no actively recruiting studies exploring this approach.

#### Toll-like receptor (TLR) agonists

Irradiation activates DCs in part via the release of TLR agonists by dying cells [74]. TLR-targeted therapy can enhance the effect of RT by improving antigen presentation by DCs. A variety of TLR agonists have been utilized in combination with RT in the preclinical and clinical setting, including IMM-101, a suspension of *Mycobacterium* that acts as a TLR-2 agonist; GLA-SE (glucopyranosyl lipid A), a TLR-4 agonist; poly-ICLC, which binds to TLR-3; Z-100, (ZERIA Pharmaceuticals), an extract obtained from *Mycobacterium tuberculosis* strain Aoyama B, which activates the innate immune system and modulates macrophage activity through an unclear mechanism; CpG (cytosine phosphate guanine), which binds to TLR9, [75] and imiquimod, which activates TLR-7 [76, 77]. A phase 3 trial for locally advanced cervical cancer randomized patients to standard of care chemoRT versus standard of care plus Z-100. There was a trend towards improved overall survival with the addition of a TLR agonist [78]. A phase 1 trial in anaplastic glioma patients administered poly-ICLC as an intramuscular injection with RT to 60 Gy total in daily 2 Gy fractions, and found a favorable overall survival rate at 1 year of 69 % [79].

CpG oligodeoxynucleotides have been shown to stimulate the anti-tumor immune response via TLR-9 mediated DC activation. Direct intratumoral injection of CpG has been studied with low-dose RT (2 Gy x 2) in a phase 1/2 study of mycosis fungoides patients, with a 50 % response rate outside of the treated site (NCT00226993) [80]. A similarly designed phase I/II study combined low dose RT (2 Gy x 2) with intratumoral injection of CpG in recurrent low-grade lymphoma patients, with promising response rate of 27 % in non-irradiated tumors (NCT00185965) [81]. Overall, this approach was well tolerated, with no treatment limiting adverse events. All patients demonstrated a tumor-specific immune response within 4 weeks. The combination approach of a second-generation TLR9 agonist (SD-101) with ipilimumab and low-dose RT is under study for low-grade lymphoma as part of a phase 1/2 dose-escalation trial (NCT01745354). At present, there are a handful active phase 1/2 studies exploring use of TLR agonists with RT in lymphoma, metastatic breast cancer and metastatic soft tissue sarcoma (Table 4).

#### Cancer vaccines

Another approach for enhancing anti-tumor immunity is vaccination through presentation of tumor antigens on recombinant viral vectors. The combination of tumor vaccines with RT has shown promise in preclinical settings and small phase 1/2 studies. Use of a poxviral-based vaccine to target PSA was combined with definitive RT in localized prostate cancer patients [82]. Vaccination was administered with GM-CSF and IL-2 as immunostimulatory adjuvants. Out of the 17 patients who completed the course of vaccinations, 13 exhibited at least 3-fold increase in PSA-specific T-cells, compared to no detectable increase with RT alone. There was also evidence of T-cell responses to prostate-associated antigens not present in the vaccine, suggestive of antigenic cascade or epitope spreading possibly induced by RT [83]. A subsequent phase 2 trial in 33 men with intermediate- to high-risk prostate cancer demonstrated a detectable PSA-specific T-cell response with vaccination [84]. Results of a phase 1 trial in pancreatic cancer (NCT00638612) were recently reported, [85] demonstrating the feasibility and safety of a combination approach incorporating recombinant viral vector vaccines with chemoRT. Similar results have been shown for a parallel approach in malignant gliomas [86]. Currently, there is an ongoing phase 3 trial randomizing men with localized prostate cancer to an adenoviral vector based PSA-vaccine (ProstAtak) with definitive RT vs placebo (NCT 01436968), and a phase 1/2 study exploring use of a similar strategy with chemoRT in locally advanced pancreatic cancer (NCT02446093).

There are multiple cancer vaccines under development or recent approval, and studies combining novel vaccines with RT to promote synergistic anti-tumor immunity are underway. Sipuleucel-T (Provenge) is a type of cancer vaccine consisting of autologous antigen-presenting cells, activated *ex vivo* and loaded with PAP (prostatic acid phosphatase) antigen, which is expressed by the majority of prostate cancer cells, and GM-CSF to promote DC maturation. Sipuleucel-T has been approved for use in metastatic, castrate-resistant prostate cancer based on phase 3 clinical trials demonstrating improved overall survival [87]. There are currently 3 active clinical trials assessing use of radiation as an immunogenic adjuvant in combination with sipuleucel-T (Table 4).

### Clinical considerations for trial evaluation

The number of clinical trials exploring use of RT with immunotherapy is rapidly increasing. It is evident that RT can expose tumor antigens to the immune system, but the optimal partnering with immunotherapies to maximize this effect remains unclear. As we move forward, there are several questions that remain unanswered, warranting discussion of relevant considerations in appropriate trial design and evaluation.

#### RT Parameters

At present, a wide range of dose and fractionation schedules are being utilized in clinical studies, and the optimal regimen to elicit an immune response remains unknown. Also unclear is whether a lower RT dose is superior to an ablative dose in this context. The appropriate selection of RT dose and fractionation is likely to be a critical determinant in successful generation of an antitumor immune response. Preclinical studies in breast and colon cancer models suggest that 3 fractions of 8 Gy, and 5 fractions of 6 Gy, remain superior to a single ablative dose of 20 Gy in combination with CTLA4 blockade to generate an abscopal response [15]. It is notable, therefore, that clinical reports of abscopal effects after CTLA4 blockade and RT have utilized similar regimens of 3 to 5 fractions, supporting these findings [26, 30]. While a fractionated approach seems superior to a single dose strategy, it remains to be determined whether ablative doses of RT could have resulted in a similar or enhanced response. Furthermore, since these studies were performed with CTLA4 blockade, further investigations need to be performed with other immune checkpoint inhibitors to determine whether similar differences will be demonstrable. Results of thoughtfully designed prospective studies will help address this question.

The size of the treatment field also influences the partnership between radiation and induction of an antitumor immune response. Circulating lymphocytes are highly sensitive to RT, with a D90 of 0.5 Gy [88]. Larger treatment fields expose a greater volume of circulating lymphocytes to RT, and also impact proliferating T-cells and potential T-cell priming in draining lymphatic regions. Even with smaller, more conformal RT fields, protracted RT regimens expose circulating lymphocytes to lymphotoxic doses, which may exhaust T-cells [89]. Strategies to reduce RT-induced lymphopenia include hypo-fractionation, reduction in treatment field size with highly conformal techniques such as SBRT/SRS and shortening of beam-on treatment times.

Another consideration when partnering RT with immunotherapy is the target site. A phase 3 trial combining a single fraction of RT to 8 Gy with CTLA-4 blockade failed to demonstrate improved overall survival, but RT was delivered on a single dose and directed towards an osseous metastasis [35]. Though abscopal responses have been seen with RT given to a bone metastasis, [90] it is notable that most published reports demonstrating abscopal responses have mainly resulted from RT directed to visceral metastases, with multiple fractions.

#### Sequencing of therapies

The optimal sequencing of RT with immunotherapy likely depends on the type of treatment used and mode of action. All reported cases of abscopal response in CTLA-4 blockade have occurred in patients who received RT concurrent with, or immediately after, therapy [15, 26, 30]. Preclinical models demonstrate that administration of RT first, followed by delayed CTLA-4 blockade, results in inferior outcomes, [15] supporting use of concurrent therapy. The majority of currently ongoing trials utilize concurrent immune checkpoint blockade with RT. An intriguing question is whether addition of other immunotherapies in a staged or concomitant fashion can improve outcomes by promoting likelihood of immunization against the tumor, and avoid T-cell exhaustion. Such an approach can incorporate RT as one of the steps in a thoughtfully designed trial.

#### Trial endpoint selection

It remains to be determined whether conventional trial endpoints such as progression-free survival, local control and disease-free survival are relevant measures of response to immunotherapy. As an example, patients randomized to sipuleucel-T, an immunotherapeutic vaccine, showed no objective response or improvement in time to disease progression, yet achieved a statistically significant 4.1 month improvement in overall survival [87]. Traditional RECIST criteria fail to account for transient increases in tumor size, which has been known to occur after immunotherapy, and can occur prior to a clinical response. Furthermore, the combination of local RT and immunotherapy can impact distant, or new lesions over the targeted lesion. In an effort to overcome these limitations, and account for changes in total tumor burden throughout the body, immune-related response criteria have been proposed [91, 92]. Improved methods of monitoring response after immunotherapy and RT are being developed, and clarity on relevant endpoint selection will be obtained as data emerges from ongoing studies. At present, overall survival, safety/toxicity and quality of life impact appear superior to traditional endpoints of disease/progression free survival and local control.

#### Patient selection

Selection of appropriate patients for trial enrollment is critical, factors such as degree of myelosuppression, overall tumor burden, neutrophil to lymphocyte ratio and prior exposure to RT and chemotherapy should be taken into consideration [43]. Depletion of immune cells decrease likelihood of immune response; thus patients with decreased lymphocyte counts due to cytotoxic chemotherapy and marrow infiltration by tumor are likely to be poor responders to treatment. Similarly, the use of larger fields during classical prolonged fractionated regimens of 30-40 fractions inevitably will reduce the availability of effector and memory cells [72]. In addition, tumor burden at the time of intervention with immunotherapy and radiation may influence outcome since patients with a significant metastatic burden did not benefit from a combined approach of CTLA-4 blockade and RT, whereas limited disease burden correlated with improved survival [35].

## Conclusion

The combination of RT with immunotherapy has exciting potential to transform cancer treatment by harnessing the immune system in a synergistic approach. While evidence in support of this combination continues to accumulate, additional data is warranted in order to determine safety and efficacy, and results of ongoing clinical trials will help address this need. In addition to this, well-designed, prospective trials will help determine the optimal dose, technique and sequencing of RT with immunotherapies. Development of biomarkers to predict treatment response to immunotherapies will help identify patients most likely to benefit from different treatments. An exciting new field of research is developing, and results of ongoing clinical trials are eagerly awaited.

## Abbreviations

GY, gray; RT, radiation therapy.

## References

[1] Formenti SC, Demaria S (2009). Systemic effects of local radiotherapy. Lancet Oncol.

[2] Grilli G, Nothdurft W, Fliedner TM (1982). Radiation sensitivity of human erythropoietic and granulopoietic progenitor cells in the blood and in the bone marrow. Int J Radiat Biol Relat Stud Phys Chem Med.

[3] Parmentier C, Morardet N, Tubiana M (1983). Late effects on human bone marrow after extended field radiotherapy. Int J Radiat Oncol Biol Phys.

[4] Barrett A (1982). Total body irradiation (TBI) before bone marrow transplantation in leukaemia: a co-operative study from the European Group for Bone Marrow Transplantation. Br J Radiol.

[5] Deeg HJ, Sullivan KM, Buckner CD, Storb R, Appelbaum FR, Clift RA, Doney K, Sanders JE, Witherspoon RP, Thomas ED (1986). Marrow transplantation for acute nonlymphoblastic leukemia in first remission: toxicity and long-term follow-up of patients conditioned with single dose or fractionated total body irradiation. Bone Marrow Transplant.

[6] Steel GG, McMillan TJ, Peacock JH (1989). The 5Rs of radiobiology. Int J Radiat Biol.

[7] Demaria S, Formenti SC (2012). Radiation as an immunological adjuvant: current evidence on dose and fractionation. Front Oncol.

[8] Golden EB, Formenti SC (2014). Is tumor (R)ejection by the immune system the “5th R” of radiobiology?. Oncoimmunology.

[9] Formenti SC, Demaria S (2013). Combining radiotherapy and cancer immunotherapy: a paradigm shift. J Natl Cancer Inst.

[10] Vanpouille-Box C, Pilones KA, Wennerberg E, Formenti SC, Demaria S (2015). In situ vaccination by radiotherapy to improve responses to anti-CTLA-4 treatment. Vaccine.

[11] Sharabi AB, Lim M, DeWeese TL, Drake CG (2015). Radiation and checkpoint blockade immunotherapy: radiosensitisation and potential mechanisms of synergy. Lancet Oncol.

[12] Golden EB, Frances D, Pellicciotta I, Demaria S, Helen Barcellos-Hoff M, Formenti SC (2014). Radiation fosters dose-dependent and chemotherapy-induced immunogenic cell death. Oncoimmunology.

[13] Filatenkov A, Baker J, Mueller AM, Kenkel J, Ahn GO, Dutt S, Zhang N, Kohrt H, Jensen K, Dejbakhsh-Jones S (2015). Ablative tumor radiation can change the tumor immune cell microenvironment to induce durable complete remissions. Clin Cancer Res.

[14] Dovedi SJ, Adlard AL, Lipowska-Bhalla G, McKenna C, Jones S, Cheadle EJ, Stratford IJ, Poon E, Morrow M, Stewart R (2014). Acquired resistance to fractionated radiotherapy can be overcome by concurrent PD-L1 blockade. Cancer Res.

[15] Dewan MZ, Galloway AE, Kawashima N, Dewyngaert JK, Babb JS, Formenti SC, Demaria S (2009). Fractionated but not single-dose radiotherapy induces an immune-mediated abscopal effect when combined with anti-CTLA-4 antibody. Clin Cancer Res.

[16] Lugade AA, Moran JP, Gerber SA, Rose RC, Frelinger JG, Lord EM (2005). Local radiation therapy of B16 melanoma tumors increases the generation of tumor antigen-specific effector cells that traffic to the tumor. J Immunol.

[17] Chen DS, Mellman I (2013). Oncology meets immunology: the cancer-immunity cycle. Immunity.

[18] Aguirre-Ghiso JA (2007). Models, mechanisms and clinical evidence for cancer dormancy. Nat Rev Cancer.

[19] Steinman RM (2012). Decisions about dendritic cells: past, present, and future. Annu Rev Immunol.

[20] Gupta A, Probst HC, Vuong V, Landshammer A, Muth S, Yagita H, Schwendener R, Pruschy M, Knuth A, van den Broek M (2012). Radiotherapy promotes tumor-specific effector CD8+ T cells via dendritic cell activation. J Immunol.

[21] Parker JJ, Jones JC, Strober S, Knox SJ (2013). Characterization of direct radiation-induced immune function and molecular signaling changes in an antigen presenting cell line. Clin Immunol.

[22] Peggs KS, Quezada SA, Allison JP (2008). Cell intrinsic mechanisms of T-cell inhibition and application to cancer therapy. Immunol Rev.

[23] Simpson TR, Li F, Montalvo-Ortiz W, Sepulveda MA, Bergerhoff K, Arce F, Roddie C, Henry JY, Yagita H, Wolchok JD (2013). Fc-dependent depletion of tumor-infiltrating regulatory T cells co-defines the efficacy of anti-CTLA-4 therapy against melanoma. J Exp Med.

[24] Ledford H (2011). Melanoma drug wins US approval. Nature.

[25] Demaria S, Kawashima N, Yang AM, Devitt ML, Babb JS, Allison JP, Formenti SC (2005). Immune-mediated inhibition of metastases after treatment with local radiation and CTLA-4 blockade in a mouse model of breast cancer. Clin Cancer Res.

[26] Postow MA, Callahan MK, Barker CA, Yamada Y, Yuan J, Kitano S, Mu Z, Rasalan T, Adamow M, Ritter E (2012). Immunologic correlates of the abscopal effect in a patient with melanoma. N Engl J Med.

[27] Stamell EF, Wolchok JD, Gnjatic S, Lee NY, Brownell I (2013). The abscopal effect associated with a systemic anti-melanoma immune response. Int J Radiat Oncol Biol Phys.

[28] Hiniker SM, Chen DS, Reddy S, Chang DT, Jones JC, Mollick JA, Swetter SM, Knox SJ (2012). A systemic complete response of metastatic melanoma to local radiation and immunotherapy. Transl Oncol.

[29] Chandra RA, Wilhite TJ, Balboni TA, Alexander BM, Spektor A, Ott PA, Ng AK, Hodi FS, Schoenfeld JD (2015). A systematic evaluation of abscopal responses following radiotherapy in patients with metastatic melanoma treated with ipilimumab. Oncoimmunology.

[30] Golden EB, Demaria S, Schiff PB, Chachoua A, Formenti SC (2013). An abscopal response to radiation and ipilimumab in a patient with metastatic non-small cell lung cancer. Cancer Immunol Res.

[31] Grimaldi AM, Simeone E, Giannarelli D, Muto P, Falivene S, Borzillo V, Giugliano FM, Sandomenico F, Petrillo A, Curvietto M (2014). Abscopal effects of radiotherapy on advanced melanoma patients who progressed after ipilimumab immunotherapy. Oncoimmunology.

[32] Golden EB, Chachoua A, Fenton-Kerimian M, Demaria S, Formenti S (2015). Abscopal responses in metastatic non-small cell lung cancer (NSCLC) patients treated on a phase 2 study of combined radiation therapy and ipilimumab: evidence for the in situ vaccination hypothesis of radiation. Int J Radiat Oncol Biol Phys.

[33] Twyman-Saint Victor C, Rech AJ, Maity A, Rengan R, Pauken KE, Stelekati E, Benci JL, Xu B, Dada H, Odorizzi PM (2015). Radiation and dual checkpoint blockade activate non-redundant immune mechanisms in cancer. Nature.

[34] Slovin SF, Higano CS, Hamid O, Tejwani S, Harzstark A, Alumkal JJ, Scher HI, Chin K, Gagnier P, McHenry MB (2013). Ipilimumab alone or in combination with radiotherapy in metastatic castration-resistant prostate cancer: results from an open-label, multicenter phase I/II study. Ann Oncol.

[35] Kwon ED, Drake CG, Scher HI, Fizazi K, Bossi A, van den Eertwegh AJ, Krainer M, Houede N, Santos R, Mahammedi H (2014). Ipilimumab versus placebo after radiotherapy in patients with metastatic castration-resistant prostate cancer that had progressed after docetaxel chemotherapy (CA184-043): a multicentre, randomised, double-blind, phase 3 trial. Lancet Oncol.

[36] Gangadhar TC, Salama AK (2015). Clinical applications of PD-1-based therapy: a focus on pembrolizumab (MK-3475) in the management of melanoma and other tumor types. Onco Targets Ther.

[37] Deng L, Liang H, Burnette B, Beckett M, Darga T, Weichselbaum RR, Fu YX (2014). Irradiation and anti-PD-L1 treatment synergistically promote antitumor immunity in mice. J Clin Invest.

[38] Sharabi AB, Nirschl CJ, Kochel CM, Nirschl TR, Francica BJ, Velarde E, Deweese TL, Drake CG (2015). Stereotactic radiation therapy augments antigen-specific PD-1-mediated antitumor immune responses via cross-presentation of tumor antigen. Cancer Immunol Res.

[39] Barcellos-Hoff MH, Akhurst RJ (2009). Transforming growth factor-beta in breast cancer: too much, too late. Breast Cancer Res.

[40] Barcellos-Hoff MH, Derynck R, Tsang ML, Weatherbee JA (1994). Transforming growth factor-beta activation in irradiated murine mammary gland. J Clin Invest.

[41] Vanpouille-Box C, Diamond JM, Pilones KA, Zavadil J, Babb JS, Formenti SC, Barcellos-Hoff MH, Demaria S (2015). TGFbeta is a master regulator of radiation therapy-induced antitumor immunity. Cancer Res.

[42] Demaria S, Ng B, Devitt ML, Babb JS, Kawashima N, Liebes L, Formenti SC (2004). Ionizing radiation inhibition of distant untreated tumors (abscopal effect) is immune mediated. Int J Radiat Oncol Biol Phys.

[43] Golden EB, Chhabra A, Chachoua A, Adams S, Donach M, Fenton-Kerimian M, Friedman K, Ponzo F, Babb JS, Goldberg J (2015). Local radiotherapy and granulocyte-macrophage colony-stimulating factor to generate abscopal responses in patients with metastatic solid tumours: a proof-of-principle trial. Lancet Oncol.

[44] Nemunaitis J (2005). Vaccines in cancer: GVAX, a GM-CSF gene vaccine. Expert Rev Vaccines.

[45] Rosenberg SA, Yang JC, Topalian SL, Schwartzentruber DJ, Weber JS, Parkinson DR, Seipp CA, Einhorn JH, White DE (1994). Treatment of 283 consecutive patients with metastatic melanoma or renal cell cancer using high-dose bolus interleukin 2. JAMA.

[46] Figlin RA, Thompson JA, Bukowski RM, Vogelzang NJ, Novick AC, Lange P, Steinberg GD, Belldegrun AS (1999). Multicenter, randomized, phase III trial of CD8(+) tumor-infiltrating lymphocytes in combination with recombinant interleukin-2 in metastatic renal cell carcinoma. J Clin Oncol.

[47] Hallahan DE, Spriggs DR, Beckett MA, Kufe DW, Weichselbaum RR (1989). Increased tumor necrosis factor alpha mRNA after cellular exposure to ionizing radiation. Proc Natl Acad Sci U S A.

[48] Reits EA, Hodge JW, Herberts CA, Groothuis TA, Chakraborty M, Wansley EK, Camphausen K, Luiten RM, de Ru AH, Neijssen J (2006). Radiation modulates the peptide repertoire, enhances MHC class I expression, and induces successful antitumor immunotherapy. J Exp Med.

[49] Seung SK, Curti BD, Crittenden M, Walker E, Coffey T, Siebert JC, Miller W, Payne R, Glenn L, Bageac A (2012). Phase 1 study of stereotactic body radiotherapy and interleukin-2--tumor and immunological responses. Sci Transl Med.

[50] McDermott DF, Regan MM, Clark JI, Flaherty LE, Weiss GR, Logan TF, Kirkwood JM, Gordon MS, Sosman JA, Ernstoff MS (2005). Randomized phase III trial of high-dose interleukin-2 versus subcutaneous interleukin-2 and interferon in patients with metastatic renal cell carcinoma. J Clin Oncol.

[51] Rosenberg SA, Yang JC, White DE, Steinberg SM (1998). Durability of complete responses in patients with metastatic cancer treated with high-dose interleukin-2: identification of the antigens mediating response. Ann Surg.

[52] Johannsen M, Spitaleri G, Curigliano G, Roigas J, Weikert S, Kempkensteffen C, Roemer A, Kloeters C, Rogalla P, Pecher G (2010). The tumour-targeting human L19-IL2 immunocytokine: preclinical safety studies, phase I clinical trial in patients with solid tumours and expansion into patients with advanced renal cell carcinoma. Eur J Cancer.

[53] Weide B, Eigentler TK, Pflugfelder A, Zelba H, Martens A, Pawelec G, Giovannoni L, Ruffini PA, Elia G, Neri D (2014). Intralesional treatment of stage III metastatic melanoma patients with L19-IL2 results in sustained clinical and systemic immunologic responses. Cancer Immunol Res.

[54] Danielli R, Patuzzo R, Di Giacomo AM, Gallino G, Maurichi A, Di Florio A, Cutaia O, Lazzeri A, Fazio C, Miracco C (2015). Intralesional administration of L19-IL2/L19-TNF in stage III or stage IVM1a melanoma patients: results of a phase II study. Cancer Immunol Immunother.

[55] Rekers NH, Zegers CM, Yaromina A, Lieuwes NG, Biemans R, Senden-Gijsbers BL, Losen M, Van Limbergen EJ, Germeraad WT, Neri D (2015). Combination of radiotherapy with the immunocytokine L19-IL2: Additive effect in a NK cell dependent tumour model. Radiother Oncol.

[56] Picozzi VJ, Abrams RA, Decker PA, Traverso W, O'Reilly EM, Greeno E, Martin RC, Wilfong LS, Rothenberg ML, Posner MC (2011). Multicenter phase II trial of adjuvant therapy for resected pancreatic cancer using cisplatin, 5-fluorouracil, and interferon-alfa-2b-based chemoradiation: ACOSOG Trial Z05031. Ann Oncol.

[57] Basu P, Jenson AB, Majhi T, Choudhury P, Mandal R, Banerjee D, Biswas J, Pan J, Rai SN, Ghim SJ (2016). Phase 2 randomized controlled trial of radiation therapy plus concurrent interferon-alpha and retinoic acid versus cisplatin for stage III cervical carcinoma. Int J Radiat Oncol Biol Phys.

[58] Hallahan DE, Vokes EE, Rubin SJ, O'Brien S, Samuels B, Vijaykumar S, Kufe DW, Phillips R, Weichselbaum RR (1995). Phase I dose-escalation study of tumor necrosis factor-alpha and concomitant radiation therapy. Cancer J Sci Am.

[59] Senzer N, Mani S, Rosemurgy A, Nemunaitis J, Cunningham C, Guha C, Bayol N, Gillen M, Chu K, Rasmussen C (2004). TNFerade biologic, an adenovector with a radiation-inducible promoter, carrying the human tumor necrosis factor alpha gene: a phase I study in patients with solid tumors. J Clin Oncol.

[60] Mundt AJ, Vijayakumar S, Nemunaitis J, Sandler A, Schwartz H, Hanna N, Peabody T, Senzer N, Chu K, Rasmussen CS (2004). A Phase I trial of TNFerade biologic in patients with soft tissue sarcoma in the extremities. Clin Cancer Res.

[61] Citrin D, Camphausen K, Wood BJ, Quezado M, Denobile J, Pingpank JF, Royal RE, Alexander HR, Seidel G, Steinberg SM (2010). A pilot feasibility study of TNFerade biologic with capecitabine and radiation therapy followed by surgical resection for the treatment of rectal cancer. Oncology.

[62] Hecht JR, Farrell JJ, Senzer N, Nemunaitis J, Rosemurgy A, Chung T, Hanna N, Chang KJ, Javle M, Posner M (2012). EUS or percutaneously guided intratumoral TNFerade biologic with 5-fluorouracil and radiotherapy for first-line treatment of locally advanced pancreatic cancer: a phase I/II study. Gastrointest Endosc.

[63] Chang KJ, Reid T, Senzer N, Swisher S, Pinto H, Hanna N, Chak A, Soetikno R (2012). Phase I evaluation of TNFerade biologic plus chemoradiotherapy before esophagectomy for locally advanced resectable esophageal cancer. Gastrointest Endosc.

[64] Seiwert TY, Darga T, Haraf D, Blair EA, Stenson K, Cohen EE, Salama JK, Villaflor V, Witt ME, Lingen MW (2013). A phase I dose escalation study of Ad GV.EGR.TNF.11D (TNFerade Biologic) with concurrent chemoradiotherapy in patients with recurrent head and neck cancer undergoing reirradiation. Ann Oncol.

[65] Herman JM, Wild AT, Wang H, Tran PT, Chang KJ, Taylor GE, Donehower RC, Pawlik TM, Ziegler MA, Cai H (2013). Randomized phase III multi-institutional study of TNFerade biologic with fluorouracil and radiotherapy for locally advanced pancreatic cancer: final results. J Clin Oncol.

[66] Moran AE, Kovacsovics-Bankowski M, Weinberg AD (2013). The TNFRs OX40, 4-1BB, and CD40 as targets for cancer immunotherapy. Curr Opin Immunol.

[67] Gough MJ, Crittenden MR, Sarff M, Pang P, Seung SK, Vetto JT, Hu HM, Redmond WL, Holland J, Weinberg AD (2010). Adjuvant therapy with agonistic antibodies to CD134 (OX40) increases local control after surgical or radiation therapy of cancer in mice. J Immunother.

[68] Yokouchi H, Yamazaki K, Chamoto K, Kikuchi E, Shinagawa N, Oizumi S, Hommura F, Nishimura T, Nishimura M (2008). Anti-OX40 monoclonal antibody therapy in combination with radiotherapy results in therapeutic antitumor immunity to murine lung cancer. Cancer Sci.

[69] Curti BD, Kovacsovics-Bankowski M, Morris N, Walker E, Chisholm L, Floyd K, Walker J, Gonzalez I, Meeuwsen T, Fox BA (2013). OX40 is a potent immune-stimulating target in late-stage cancer patients. Cancer Res.

[70] Kovacsovics-Bankowski M, Vercellini J, Crittenden M, Lary S, Curti B, Weinberg A. Phase i/ii clinical trial of anti-OX40, radiation and cyclophosphamide in patients with prostate cancer. J Immunother Cancer. 2013;1(Suppl 1):P255.

[71] Finkelstein SE, Rodriguez F, Dunn M, Farmello MJ, Smilee R, Janssen W, Kang L, Chuang T, Seigne J, Pow-Sang J (2012). Serial assessment of lymphocytes and apoptosis in the prostate during coordinated intraprostatic dendritic cell injection and radiotherapy. Immunotherapy.

[72] Chi KH, Liu SJ, Li CP, Kuo HP, Wang YS, Chao Y, Hsieh SL (2005). Combination of conformal radiotherapy and intratumoral injection of adoptive dendritic cell immunotherapy in refractory hepatoma. J Immunother.

[73] Raj S, Bui MM, Springett G, Conley A, Lavilla-Alonso S, Zhao X, Chen D, Haysek R, Gonzalez R, Letson GD (2015). Long-term clinical responses of neoadjuvant dendritic cell infusions and radiation in soft tissue sarcoma. Sarcoma.

[74] Gallucci S, Lolkema M, Matzinger P (1999). Natural adjuvants: endogenous activators of dendritic cells. Nat Med.

[75] Mason KA, Ariga H, Neal R, Valdecanas D, Hunter N, Krieg AM, Whisnant JK, Milas L (2005). Targeting toll-like receptor 9 with CpG oligodeoxynucleotides enhances tumor response to fractionated radiotherapy. Clin Cancer Res.

[76] Dewan MZ, Vanpouille-Box C, Kawashima N, DiNapoli S, Babb JS, Formenti SC, Adams S, Demaria S (2012). Synergy of topical toll-like receptor 7 agonist with radiation and low-dose cyclophosphamide in a mouse model of cutaneous breast cancer. Clin Cancer Res.

[77] Dovedi SJ, Melis MH, Wilkinson RW, Adlard AL, Stratford IJ, Honeychurch J, Illidge TM (2013). Systemic delivery of a TLR7 agonist in combination with radiation primes durable antitumor immune responses in mouse models of lymphoma. Blood.

[78] Sugiyama T, Fujiwara K, Ohashi Y, Yokota H, Hatae M, Ohno T, Nagai Y, Mitsuhashi N, Ochiai K, Noda K (2014). Phase III placebo-controlled double-blind randomized trial of radiotherapy for stage IIB-IVA cervical cancer with or without immunomodulator Z-100: a JGOG study. Ann Oncol.

[79] Butowski N, Lamborn KR, Lee BL, Prados MD, Cloughesy T, DeAngelis LM, Abrey L, Fink K, Lieberman F, Mehta M (2009). A North American brain tumor consortium phase II study of poly-ICLC for adult patients with recurrent anaplastic gliomas. J Neurooncol.

[80] Shi Y, Evans JE, Rock KL (2003). Molecular identification of a danger signal that alerts the immune system to dying cells. Nature.

[81] Apetoh L, Ghiringhelli F, Tesniere A, Obeid M, Ortiz C, Criollo A, Mignot G, Maiuri MC, Ullrich E, Saulnier P (2007). Toll-like receptor 4-dependent contribution of the immune system to anticancer chemotherapy and radiotherapy. Nat Med.

[82] Gulley JL, Arlen PM, Bastian A, Morin S, Marte J, Beetham P, Tsang KY, Yokokawa J, Hodge JW, Menard C (2005). Combining a recombinant cancer vaccine with standard definitive radiotherapy in patients with localized prostate cancer. Clin Cancer Res.

[83] Vanderlugt CJ, Miller SD (1996). Epitope spreading. Curr Opin Immunol.

[84] Fujita T, Teh BS, Timme TL, Mai WY, Satoh T, Kusaka N, Naruishi K, Fattah EA, Aguilar-Cordova E, Butler EB (2006). Sustained long-term immune responses after in situ gene therapy combined with radiotherapy and hormonal therapy in prostate cancer patients. Int J Radiat Oncol Biol Phys.

[85] Aguilar LK, Shirley LA, Chung VM, Marsh CL, Walker J, Coyle W, Marx H, Bekaii-Saab T, Lesinski GB, Swanson B (2015). Gene-mediated cytotoxic immunotherapy as adjuvant to surgery or chemoradiation for pancreatic adenocarcinoma. Cancer Immunol Immunother.

[86] Chiocca EA, Aguilar LK, Bell SD, Kaur B, Hardcastle J, Cavaliere R, McGregor J, Lo S, Ray-Chaudhuri A, Chakravarti A (2011). Phase IB study of gene-mediated cytotoxic immunotherapy adjuvant to up-front surgery and intensive timing radiation for malignant glioma. J Clin Oncol.

[87] Kantoff PW, Higano CS, Shore ND, Berger ER, Small EJ, Penson DF, Redfern CH, Ferrari AC, Dreicer R, Sims RB (2010). Sipuleucel-T immunotherapy for castration-resistant prostate cancer. N Engl J Med.

[88] Nakamura N, Kusunoki Y, Akiyama M (1990). Radiosensitivity of CD4 or CD8 positive human T-lymphocytes by an in vitro colony formation assay. Radiat Res.

[89] Yovino S, Kleinberg L, Grossman SA, Narayanan M, Ford E (2013). The etiology of treatment-related lymphopenia in patients with malignant gliomas: modeling radiation dose to circulating lymphocytes explains clinical observations and suggests methods of modifying the impact of radiation on immune cells. Cancer Invest.

[90] Ohba K, Omagari K, Nakamura T, Ikuno N, Saeki S, Matsuo I, Kinoshita H, Masuda J, Hazama H, Sakamoto I (1998). Abscopal regression of hepatocellular carcinoma after radiotherapy for bone metastasis. Gut.

[91] Wolchok JD, Hoos A, O'Day S, Weber JS, Hamid O, Lebbe C, Maio M, Binder M, Bohnsack O, Nichol G (2009). Guidelines for the evaluation of immune therapy activity in solid tumors: immune-related response criteria. Clin Cancer Res.

[92] Hoos A, Eggermont AM, Janetzki S, Hodi FS, Ibrahim R, Anderson A, Humphrey R, Blumenstein B, Old L, Wolchok J (2010). Improved endpoints for cancer immunotherapy trials. J Natl Cancer Inst.

